# Developmentally Delayed Male with Mincer Blade Obstructing the Oesophagus for a Period of Time Suspected to Be 6 Months

**DOI:** 10.1155/2015/139647

**Published:** 2015-07-08

**Authors:** Christian Grønhøj Larsen, Birgitte Charabi

**Affiliations:** Department of Otorhinolaryngology, Head and Neck Surgery and Audiology, Copenhagen University Hospital (Rigshospitalet), 2100 Copenhagen, Denmark

## Abstract

*Introduction*. Sharp, retained foreign bodies in the oesophagus are associated with severe complications. Developmentally delayed patients are especially subject to foreign objects. We describe a 37-year-old, developmentally delayed male with a mincer blade obstructing the oesophagus. Six months prior to surgical intervention, the patient was hospitalized in a condition of sepsis and pneumonia where the thoracic X-ray reveals a foreign body in the proximal oesophagus. When rehospitalized 6 months later, a mincer blade of the type used in immersion blenders was surgically removed. During these 6 months the patient's main symptoms were dysphagia, weight loss, and diarrhoea. When developmentally delayed patients present with dysphagia, we strongly encourage the awareness of the possible presence of foreign bodies. To our knowledge this is the first reported case of a mincer blade in the oesophagus.

## 1. Introduction

Chronically retained foreign bodies (FBs) are common in children but rare in adults [1, 2]. For developmentally delayed adults, especially those in the subgroup with pica, FBs can not only be the cause of severe morbidity such as obstruction, bleeding, and perforation but also be lethal because the patient's inability to communicate the symptoms makes it difficult to arrive at an accurate diagnosis. Ingestion of a sharp FB is associated with a high risk of morbidity as a result of the possible perforation of the gastrointestinal tract. Early diagnosis and adequate management are imperative for the prevention of serious complications.

## 2. Case Report

**Figure 1:**
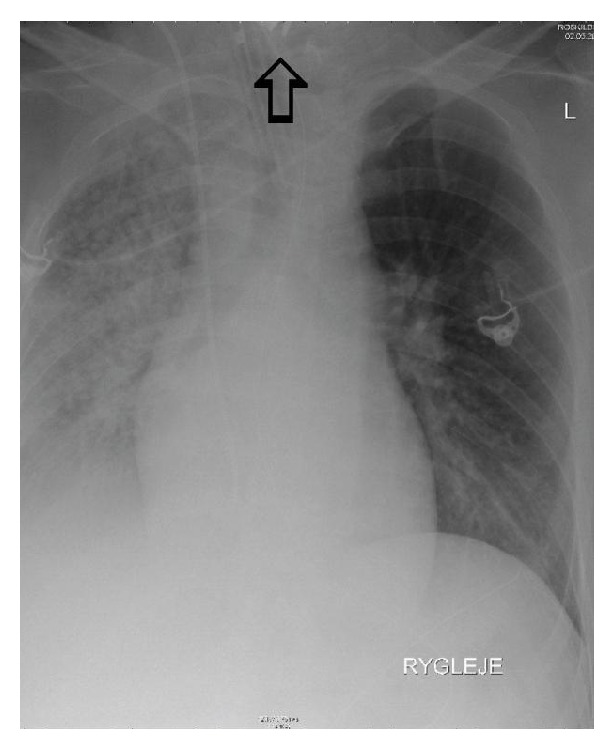
Chest X-ray 6 months prior to admittance to our department. Severe right-sided pneumonia and a suspected foreign object in the oesophagus.

**Figure 2:**
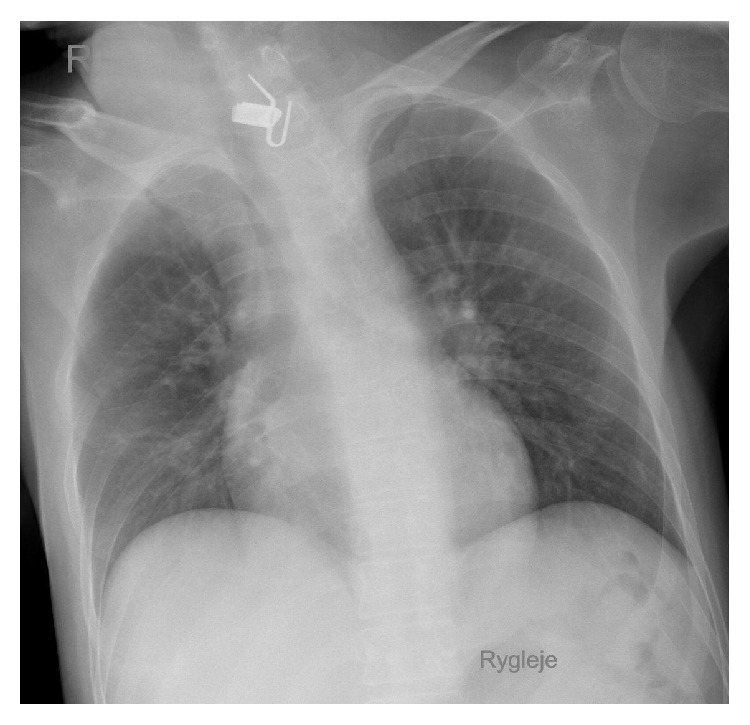
Chest X-ray.

**Figure 3:**
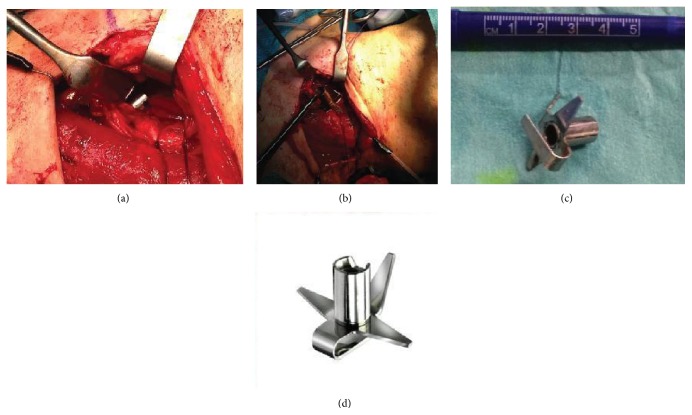
(a, b, c) Perioperative image, (d) Bamix, Mézières, Schweiz.

A 37-year-old male with the mental age of a 1-year-old was referred to our department. Six months earlier, the patient had been admitted to a neighbouring hospital with aspiration pneumonia in a condition of sepsis and complaining of dysphagia. A chest X-ray was performed (Figure 1). The patient was treated with antibiotics and discharged on the 23th day, returning to his nursing home in good clinical condition, but with the dysphagia unchanged. During the 6 months following his initial hospitalization the patient's loss of 20 kilograms, persistent diarrhoea, and aversion to solid foods led to his readmittance. Because of the severe dysphagia the patient was referred to the local ear, nose, and throat department, where a cervical X-ray (Figure 2) visualized a FB. Oesophagoscopy revealed a shiny, asymmetric, metallic structure located in the proximal oesophagus. Despite several attempts, the object was not retractable. Therefore, open oesophagotomy was performed on level C4-C5, revealing severe inflammation and fibrosis in the tissue surrounding the oesophagus. After access to the oesophageal lumen was attained, a mincer blade of the type used in immersion blenders was surgically removed (Figure 3). The blades of the mincer had penetrated all layers of the oesophagus. After removal of the blade, direct suture without reinforcements was performed to close the oesophagus. The patient was treated with antibiotics, received parenteral nutrition for 7 days, and was discharged on the 10th day. Staff at the nursing home where the patient resides recalled that a mincer blade had gone missing.

## 3. Discussion

Developmentally delayed persons with pica are especially subject to FBs in the oesophagus [3]. Objects often impact here because of the passive, distensible, and accommodating nature of the organ. Our report documents the case of a developmentally delayed male with a mincer blade lodged in the proximal oesophagus. When assessing the thoracic X-ray 6 months prior to admittance it is likely that the object was visible in the top edge of the picture with the characteristic asymmetric sharp edges of the mincer pointing out (Figure 1). This might explain the patient's symptoms of prolonged dysphagia, weight loss, and diarrhoea.

Similar cases of chronically retained FBs have been reported with successful outcomes, [1, 4, 5] although most objects usually pass spontaneously, and less than 1% need surgical removal [4]. Early diagnosis and management might prevent the serious complications of penetration, infection, and necrosis [6]. When FBs are suspected, the appropriate diagnostic approach should be biplane X-rays, which reveal the location, size, and shape of possible objects, alternatively a CT scan of the esophagus [5]. Direct vision such as oesophagoscopy or laryngoscopic-aided views are highly useful means of obtaining additional diagnostic information. Moreover, endoscopy may also provide the treatment as many objects can be extracted endoscopically [7]. Migratory oesophageal FBs are particularly rare [1].

Sharp objects most commonly lodge at the upper level of the oesophagus due to penetration into the upper oesophageal sphincter. Whenever possible, FBs in the oesophagus should be sought and removed endoscopically [5]; however, if surgical removal is unavoidable, it must be performed without delay, in order to avoid potentially severe complications, such as mediastinitis, fistula, pneumothorax, respiratory distress, retropharyngeal abscess, and stricture. Nonsharp objects lodged in the oesophagus for more than 24 hours should also be removed endoscopically. However, if they remain for more than a week, there is significant risk of erosion into surrounding structures, and surgical backup should always be available [1, 5].

When dealing with the severely mentally retarded group of patients, common symptoms such as dysphagia, odynophagia, coughing, choking, and haematemesis may be compromised. For this reason, information about the patient's general condition, appetite, and bowel habits is vital. If the general condition of a patient with these symptoms worsens and persists over a long period of time, FBs should always be considered and biplane X-rays of the upper oesophagus must be performed without delay [8].

Earlier cases of oesophageal FBs involving developmentally challenged individuals have included impacted dentures [3], coins [9], and food items, but this is the first reported case of a mincer blade from an immersion blender lodging in the oesophagus.

A thorough elucidation of developmentally delayed patients with dysphagia is imperative, and the presence of foreign objects in the pharynx, oesophagus, or intestine must always be considered when such patients present with a declining general condition, reduced appetite, abnormal bowel movements, or dysphagia. Biplane X-rays of the oesophagus and endoscopy should be performed initially and, particularly in cases involving sharp FBs, surgery must not be delayed.

## 4. Conclusion

We know the following:

- The elucidation of developmentally delayed patients is difficult.
- This specific patient-population is especially subject to foreign bodies in the oesophagus.

This study adds the following:

- When developmentally delayed patients present with dysphagia, altered bowel movements, and declining general condition, foreign bodies must be considered.
- Biplane X-rays and endoscopy are vital initial steps.
